# Methamphetamine Self-Administration Elicits Sex-Related Changes in Postsynaptic Glutamate Transmission in the Prefrontal Cortex

**DOI:** 10.1523/ENEURO.0401-18.2018

**Published:** 2019-01-28

**Authors:** Jose Ignacio Pena-Bravo, Rachel Penrod, Carmela M. Reichel, Antonieta Lavin

**Affiliations:** 1 Department of Neuroscience, Medical University of South Carolina, Charleston, SC 29425

**Keywords:** gluatamte, metampetamine, NMDA, prefrontal cortex, sex

## Abstract

Preclinical and clinical research has shown that females are more vulnerable to the rewarding effects of stimulants, and it has been proposed that estrogens may play a role in this enhanced sensitivity; however sex differences in methamphetamine (METH)-induced neuroplasticity have not been explored. To address this gap in knowledge, we recorded from the prelimbic area of the prefrontal cortex (PL-PFC) of male and female rats following long access METH self-administration (SA) and investigated the resulting long-term synaptic neuroadaptations. Males and females took similar amounts of METH during SA; however, female rats exhibit significant synaptic baseline differences when compared to males. Furthermore, females exhibited a significant increase in evoked excitatory currents. This increase in evoked glutamate was correlated with increases in NMDA currents and was not affected by application of a GluN2B selective blocker. We propose that METH SA selectively upregulates GluN2B-lacking NMDA receptors (NMDAR) in the PFC of female rats. Our results may provide a mechanistic explanation for the sex differences reported for METH addiction in females.

## Significance Statement

The rate of methamphetamine (METH) addiction is second only to heroin, and use trends are on the rise. Like other drugs of abuse, there are known sex differences in METH addiction; however, whether these differences result from sex differences in pharmacokinetics, neurophysiology, and/or drug-induced neuroplasticity has not been established. Here we assessed METH self-administration (SA)-induced neuroadaptations in synaptic physiology using electrophysiological measures in prefrontal cortical (PFC) brain slices of male and female rats. We are the first to report, to our knowledge, that cortical baseline and METH-induced synaptic responses are different between male and female rats. Our results may provide a mechanistic explanation for the sex differences reported for METH addiction in females and highlight the possibility that sex-specific treatment strategies may be required to address METH-induced neuroplasticity.

## Introduction

Methamphetamine (METH) addiction is second only to heroin, and use trends are on the rise in North America (UNODC, 2017). Chronic METH use results in dysregulated cortical function and ensuing cognitive sequelae contributing to the chronic relapsing nature of METH addiction (for review, see Bernheim et al., 2017). The underlying pathology of addiction and relapse clearly implicate changes in synaptic plasticity within the reward circuit as one of the fundamental mechanisms underlying this disorder (Luthi and Luscher, 2014). The prefrontal cortex (PFC) exerts top-down control over behavior and sends glutamatergic projections to the nucleus accumbens (among other areas) and dysfunction of the medial PFC (mPFC) is credited with the loss of control over drug taking behavior (Goldstein and Volkow, 2011).

While considerable advancements have been made to understand the circuits underlying METH addiction, relapse, and cognitive dysfunction, very little is known about the synaptic mechanisms in play. Experimenter delivered METH (1 mg/kg, 7 d) decreases calcium currents and excitatory transmission in the mPFC 4 d after drug treatment (González et al., 2016). Additionally, we have shown that long-access METH self-administration (SA; 6 h/d) increases the proportion of burst firing pyramidal cells compared to tonic firing cells in mPFC (Parsegian et al., 2011); however, it was unknown whether this increased burst firing results from presynaptic and/or postsynaptic changes. In a recent study, we found that long-access METH SA decreases AMPA/NMDA ratio in pyramidal neurons of the mPFC, accompanied by an increase in NMDA currents and surface expression of the GluN2B subunit of the NMDA receptor (NMDAR; Mishra et al., 2017). Together, these results show that METH elicits multiple forms of long-term synaptic changes in the PFC, suggesting that altered synaptic function is one of the fundamental mechanisms affected by this drug.

While the aforementioned studies have all been conducted in male rats, there are known sex differences in METH SA behavior. Females are reported to acquire METH SA faster than males (Roth and Carroll, 2004), take more drug (Roth and Carroll, 2004; Reichel et al., 2012; Bernheim et al., 2017), and have greater motivation to seek the drug (Roth and Carroll, 2004; Cox et al., 2013, 2017). Male and female rats typically reinstate to METH cues in a similar manner (Bernheim et al., 2017; Cox et al., 2017), but females reinstate to a greater magnitude than males in response to priming injections of the drug (Reichel et al., 2012; Cox et al., 2013). Furthermore, in an incubation of METH craving model, females seek drug to a greater degree than males following 14 d of abstinence (Ruda-Kucerova et al., 2015). However, following a period of voluntary abstinence, male and female rats equivalently abstain for METH when given a choice between drug and food (Venniro et al., 2017). Whether these differences result from sex differences in METH pharmacokinetics (Milesi-Hallé et al., 2007, 2015) or METH-induced neuroplasticity has not been established.

To address this question, we recorded from the prelimbic area of the PFC (PL-PFC) of male and female rats following long access METH SA. We report that males and females took similar amounts of METH during SA; however, female rats exhibit synaptic differences when compared to males and METH SA elicited significant changes in NMDA kinetics in female rats. Furthermore, we found a significant increase in evoked glutamate in females, that was correlated with increases in GluN2B-lacking NMDA currents. These results provide important insight into a possible mechanism underlying the increased susceptibility to METH addiction in females and highlight a possible sex-specific plasticity for future pharmacotherapeutic targeting.

## Materials and Methods

### Animal housing

Age-matched Sprague Dawley male (225–250 g) and female (200–225 g) rats (*n* = 16; eight males and eight females) were obtained from Charles River and individually housed on 12/12 h reversed light/dark cycle with free access to water and food, until the beginning of SA (see Catheter implantation surgery). During the SA sessions, rats were provided 15–30 g of chow per day with free access to drinking water in their respective home cage. All procedures were conducted in accordance with the Guide for the Care and Use of Laboratory Rats (Institute of Laboratory Animal Resources on Life Sciences, National Research Council, 2011) and were approved by the Institutional Animal Care and Use Committee of the University.

### Catheter implantation surgery

Rats were anesthetized with intraperitoneal injections of ketamine (66 mg/kg; Vedco Inc.), xylazine (1.3 mg/kg; Lloyd Laboratories), and equithesin (0.5 ml/kg; 4 mg/kg sodium pentobarbital, 17 mg/kg chloral hydrate, and 21.3 mg/kg magnesium sulfate heptahydrate dissolved in 44% propylene glycol and 10% ethanol solution). Ketorolac (2.0 mg/kg, i.p.; Sigma) was given just before surgery as an analgesic. One end of a SILASTIC catheter was inserted 33 mm into the external right jugular and secured with 4.0 silk sutures. The other end ran subcutaneously and exited from a small incision just below the scapula. That end was attached to an infusion harness (Instech Solomon) that provided access to an external port for intravenous drug delivery. Following that surgical procedure, rats were given a subcutaneous injection of an antibiotic solution cefazolin (10 mg/0.1 ml; Schein Pharmaceuticals) and were allowed to recover for 5 d.

### METH SA

SA occurred in chambers (30 × 20 × 20 cm, Med Associates) housed inside sound-attenuating cubicles that were fitted with a fan, metal arm, and spring leash attached to a swivel (Instech); two retractable levers; two stimulus lights; a speaker for tone delivery; and a house light. Tygon tubing extended through the leash and connected to a 10-ml syringe mounted on an infusion pump outside the cubicle. After 5 d of recovery, rats were assigned to METH or saline control groups. METH hydrochloride (Sigma), dissolved in sterile saline, was administered daily during 6-h sessions for 14 d. Control animals self-administered saline. The house light signaled the beginning of a session and remained on throughout the session. A response on the active lever resulted in a 2-s infusion of METH (0.05 mg/kg per infusion) or saline and presentation of a stimulus complex that consisted of a 5-s tone (78 dB, 4.5 kHz) and a white stimulus light over the active lever, followed by a 20-s time out. Responses during the time out and on the inactive lever were recorded, but they had no scheduled consequences. Following SA, rats were placed on home cage abstinence for 9–14 d, then they were killed for end point measures of electrophysiology.

### Brain slice preparation

Rats were deeply anesthetized using isoflurane (Minrad Inc.) and the brain was quickly isolated following decapitation. Coronal slices containing mPFC (300 µm) were cut in ice-cold high-sucrose solution containing: 200 mM sucrose, 1.9 mM KCl, 1.2 mM Na_2_HPO_4_, 33 mM NaHCO_3_, 6 mM MgCl_2_, 0.5 mM CaCl_2_, 10 mM D-glucose, and 0.4 mM ascorbic acid using a Leica VT 1200 S vibratome. Slices were incubated at 31°C for at least 1 h before recordings; the incubation medium contained: 120 mM NaCl, 2.5 mM KCl, 1.25 mM NaH_2_PO_4_, 25 mM NaHCO_3_, 4 mM MgCl_2_, 1 mM CaCl_2_, 10 mM D-glucose, and 0.4 mM ascorbic acid, aerated with 5% CO_2_/95% O_2_. Following incubation, slices were transferred to a submerged chamber and superfused at room temperature with oxygenated artificial CSF (aCSF): 126 mM NaCl, 2.5 mM KCl, 1.4 mM NaH_2_PO_4_, 25 mM NaHCO_3_, 2.0 mM CaCl_2_, 1.3 mM MgCl_2_, 10 mM D-glucose, and 0.4 mM ascorbic acid.

### Voltage clamp recordings

Electrophysiological recordings were obtained with a Multiclamp 700B amplifier (Molecular Devices). Signals were low-pass filtered at 3 kHz and digitized at 10 kHz. Data were stored on a PC for off-line analysis. Data acquisition was performed using Axograph-X software (J. Clements). Analysis of spontaneous EPSCs (sEPSCs) and evoked EPSCs (eEPSCs) peak amplitude data were done in Mini Analysis (v6.0.7; Synaptosoft). For voltage-clamp recordings, electrodes (2.5–3.5 MΩ resistance *in situ*) were filled with a solution containing: 130 mM CsCl, 10 mM HEPES, 2 mM MgCl_2_, 0.5 mM EGTA, 2 mM Na_2_ATP, 0.3 mM Na-GTP, 2 mM QX-314, 10 mM phosphocreatine, and 285 mOsmol. Briefly, membrane potential was held at −70 mV and GABA-mediated events were pharmacologically isolated by adding picrotoxin (50 μM) to the bath. Series resistance (Rs) was continuously monitored by applying a small hyperpolarizing voltage step (–5 mV, 50 ms), and recordings that exhibit changes in baseline Rs > 30% were discarded. sEPSC recordings consisted of five sweeps/10-s-long recordings that were analyzed for amplitude and frequency of detected events. After recordings sEPSCs, the membrane potential was shifted to +40 mV and 10 µM CNQX (non-NMDAR blocker) was added for at least 5 min to isolate NMDA-mediated responses. Eight to 10 isolated NMDA responses were recorded. The AMPA current was obtained by digital subtraction (*I*_Total_ − *I*_NMDA_), and the ratio was calculated with the following formula: *I*_AMPA_/*I*_Total_ − *I*_AMPA_. The cells recorded did not differ statistically in their mean capacitance (saline females: 0.52 ± 0.39 pF; METH females: 0.84 ± 1.4 pF; saline males: 0.32 ± 0.28 pF; METH males: 0.89 ± 0.35 pF, Axograph). After recording AMPA/NMDA ratio, the membrane potential was clamped at –40 mV, to alleviate the Mg+ blockade, and 8–10 baseline evoked potentials were recorded before adding the GluN2B blocker Ro256981 (1 μm; Tocris) and after 5 min evoked potentials recordings were resumed for 8–10 events. For sEPSC and eEPSC kinetics, we used Minianalysis, and the parameters for detection were: threshold: five times the root mean square of noise; period to search a local maximum: 7000 µs; time before a peak: 25,000 µs; period to search a decay: 150,000 µs; function of peak to find a decay time: 0.10; and period to average a baseline: 2000 µs. All detected events per cell were used to obtain average rise (ms), decay (ms), and area (pA*mV) for all experimental groups. All recordings were obtained from pyramidal neurons in Layer V or VI of the prelimbic mPFC which were identified using infrared-differential interference contrast optics and video microscopy. We recorded four rats per group. All recordings were performed at the same time of the day and the experimenter was blind to the animal treatment.

To assess whether METH SA elicited changes in release probability or the number of releases sites, we assessed the coefficient of variation (CV) of sEPSCs (Choi and Lovinger, 1997).

### Data analysis

#### METH SA

Lever presses and drug intake were the primary dependent measures during SA. Independent variables included group (METH and saline), sex (males and females), lever (active and inactive), and day (14 d of SA). Data were analyzed with ANOVA. Lever presses were analyzed with a three-way ANOVA including group, lever, and day as variables separately for males and females. A significant three-way interaction was deconstructed with follow-up two-way ANOVAs to compare lever responding at the different levels of the group independent variable. Males and females were directly compared on METH intake with a two-way ANOVA with sex and day as the independent variables. Sidak’s Holmes adjustments were used to control for family-wise error. All data were analyzed and plotted using GraphPad Prism version 7.0 (GraphPad Software, Inc).

#### Electrophysiology

Amplitude, frequency, rise time area under the curve and decay time (sEPSCs and eEPSCs) were the dependent measures for most electrophysiology experiments. Independent variables of sex (males and females) and drug condition (METH and saline) were examined using a 2 × 2 between subjects ANOVA, and interactions were followed by Tukey’s *post hoc* comparisons for between subjects variable and Sidak’s Holmes for within subject variables. Additionally, following the guidance of our statisticians in the Department of Biostatistics, Epidemiology, and Research Design at Medical University of South Carolina, we conducted pairwise contrasts since ANOVA can only test one hypothesis (the global test) and may miss some meaningful pairwise differences between groups. sEPSCs and eEPSCs were analyzed using Minianalysis software by Synaptosoft version 6.0.7. The threshold for noise was selected as five times the RMS noise, measured in a 125-ms episode. CV was calculated as δ/μ, where δ is the Standard Deviation of the peak sEPSC amplitude and μ is the mean sEPSC amplitude. All data were analyzed and plotted using GraphPad Prism version 7.0 (GraphPad Software, Inc).

## Results

### Metamphetamine Self-Administration

**Figure 1.**
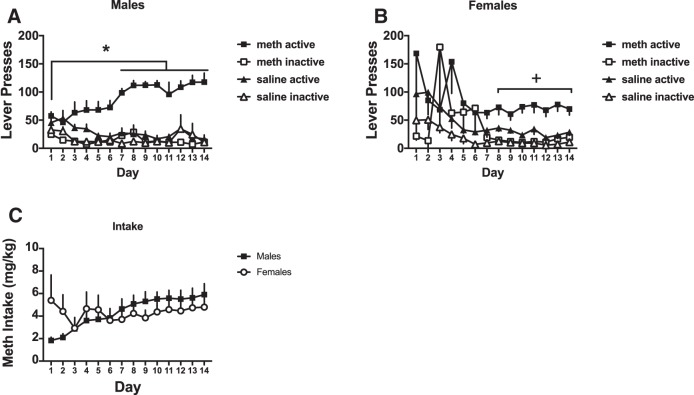
Lever presses and METH intake for male and female rats. ***A***, Male rats escalated active lever presses over time and responded more on the active lever relative to saline self-administering rats and the inactive lever. ***B***, Female rats pressed the active lever more relative to saline self-administering rats and the inactive lever during the last week of SA. ***C***, Male and female rats took similar amount of METH throughout SA; *significantly different from day 1 of SA (*p* < 0.05); +, significantly different from inactive lever responding. All the figures represent average ± SEM.

During the SA period, we assessed active and inactive lever responding in METH and saline-trained rats across 14 d of SA (*n* = 4/group). In male rats (Fig. 1*A*), there was a significant three-way interaction between group (METH vs saline), lever (active vs inactive), and day (*F*_(13,13)_ = 3.02, *p* < 0.05; Fig. 1*A*). Lever responses over day were analyzed at the two levels of the independent variable group. For METH rats there was a lever by day interaction (two-way ANOVA, *F*_(13,78)_ = 5.81, *p* < 0.05) with *post hoc* comparisons showing that active lever responding increased on days 8–14 relative to day 1 (Sidak–Holm *post hoc*, *p* < 0.05). Further responding on the active lever was significantly above the inactive lever on all 14 d (Sidak–Holm *post hoc*, *p* < 0.05). For saline rats, there was no significant lever by day interaction, but there was a significant main effect of day (*F*_(13,78)_ = 3.12, *p* < 0.05). *Post hoc* comparisons over day did not reveal any significant differences between days.

In female rats (Fig. 1*B*), the three-way ANOVA was not significant, nor were the resulting two-way ANOVAs. There were however, main effects of group (*F*_(13,13)_ = 14.53, *p* < 0.05), lever (*F*_(13,13)_ = 21.03, *p* < 0.05), and day (*F*_(13,13)_ = 2.28, *p* < 0.05). To determine whether METH females acquired lever discrimination, we conducted a two-way ANOVA with lever and day as the independent variables. There was a significant main effect of lever (*F*_(1,6)_ = 23.89, *p* < 0.05) with active lever responses being higher than inactive responses.

The amount of METH taken by male and female rats over the 14 d of SA did not differ (Fig. 1*C*). There was a significant interaction between sex and day (*F*_(13,78)_ = 2.2, *p* < 0.017), but *post hoc* comparisons did not reveal significant group differences. The main effect of day was significant (*F*_(13,78)_ = 2.27, *p* < 0.013), but the main effect of sex was not significant.

### Synaptic activity

To determine whether a history of METH SA produced sex-specific forms of synaptic plasticity, we examined changes in synaptic activity in deep layers (Layer V/VI) pyramidal neurons in the prelimbic PFC. Following 14 d of METH SA and 9–14 d of withdrawal, we assessed spontaneous and evoked synaptic activity (amplitude, frequency, kinetics) with sex and treatment (METH vs saline) as factors.

**Figure 2.**
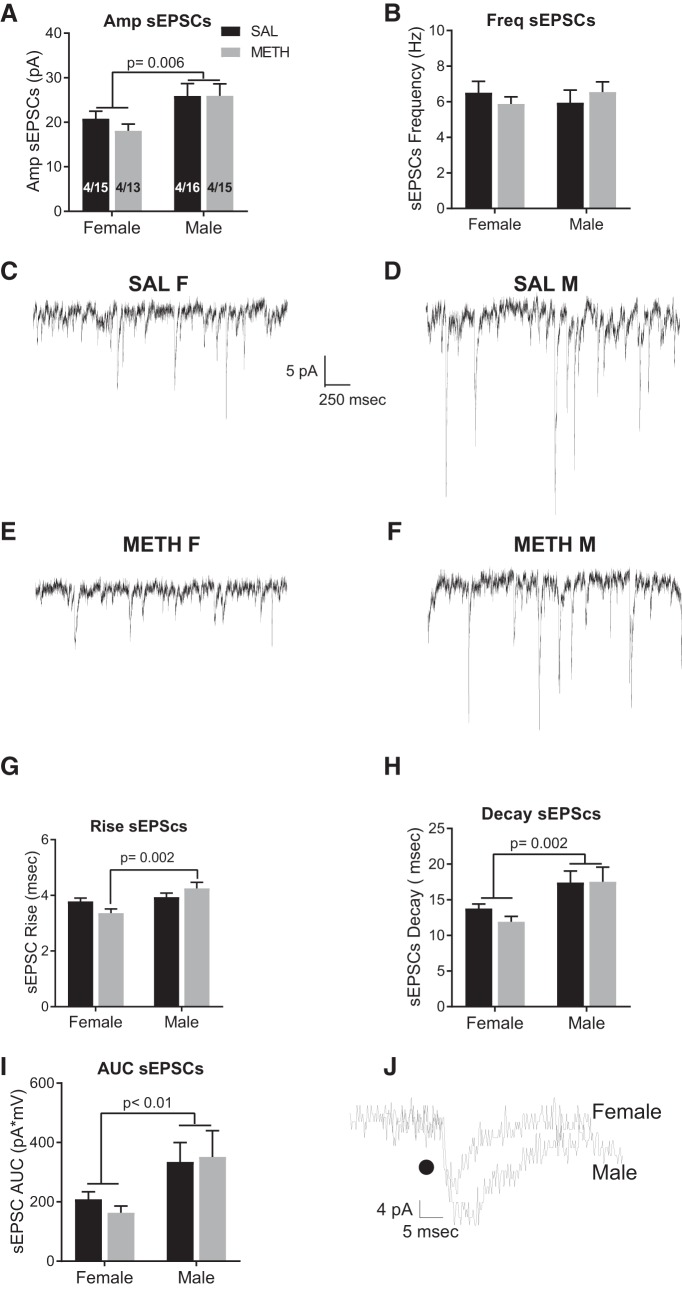
Baseline sex differences in spontaneous excitatory activity. ***A***, Female rats exhibited significantly lower amplitude of sEPSCs when compared to male rats; but ***B*** shows there were no significant differences in the frequency of sEPSCs between males or females and self-administered METH or saline. ***C–F***, Representative traces of sEPSCs in saline females (SAL F), saline males (SAL M), METH females, and METH male rats. ***G***, Female rats had a significantly faster rise of sEPSCs relative to males and (***H***) female rats exhibited a significant faster decay of sEPSCs. ***I***, Relative to males, female rats had a significant decrease in the AUC of sEPSCs. ***J***, Representative traces illustrating sex differences in kinetics of sEPSCs. The numbers in the bars represent the number of rats/number of cells.

Females had significantly lower sEPSC amplitude relative to males, indicated by a main effect of sex (*F*_(1,59)_ = 7.99, *p* = 0.006; Fig. 2*A*). There was no significant interaction or main effect of drug treatment. There we no differences in frequency of sEPSCs (Fig. 2*B*). Representative traces are depicted in Figure 2*C–F*. Measures of the sEPSCs CV confirm that METH SA did not elicit changes in probability of release of glutamate (female saline: 0.89 ± 0.11; female METH: 0.85 ± 0.14; male saline: 0.83 ± 0.05; male METH: 0.91 ± 0.01; data not shown). Male rats had significantly slower sEPSC rise and decay, and a larger area under the curve (AUC), compared to females. Specifically, for the rise of sEPSCs, there was a significant interaction between sex and treatment (*F*_(1,59)_ = 5.0, *p* = 0.002; Fig. 2*G*). Female METH rats have a significantly faster rise than male METH rats (Tukey’s, *p* = 0.002). On the decay of sEPSCs, there was a main effect of sex (*F*_(1,59)_ = 10.34, *p* = 0.002; Fig. 2*H*). The interaction and main effect of drug were not significant. Similarly for the AUC, there was a main effect of sex (*F*_(1,59)_ = 7.0, *p* = 0.01; Fig. 2*I*) with females exhibiting reduced AUC, consistent with the faster rise and decay times. The interaction and main effect of drug were not significant. Figure 2*J* depicts representative traces from male and female rats.

In summary, female rats exhibit significant decreases in basal glutamatergic excitatory strength when compared to males.

### Evoked EPSCs

**Figure 3.**
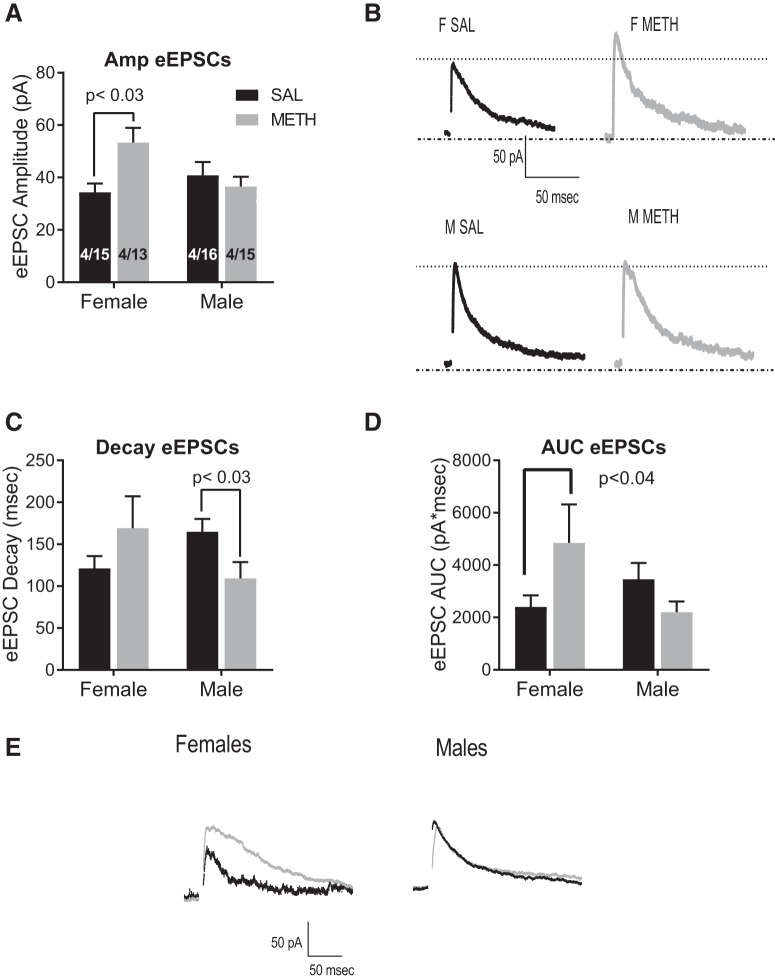
Effects of METH SA on eEPSCs. ***A***, Female rats that self-administered METH had increased eEPSCs amplitude than saline control females; whereas there were no differences in males. ***B***, Representative traces of eESPC’s amplitude. ***C***, When analyzing the decay of eEPSCs, we found a significant interaction. In females the decay was not significantly different but in males, METH SA rats show a significantly faster decay than SAL counterparts (*t* test, *p* = 0.01). ***D***, Analysis of the AUC also shows a significant interaction and a significant increase in the AUC of female METH SA rats. ***E***, Representative traces showing the kinetics of eEPSCs. Each trace is the average of 10 events.

To investigate changes in evoked glutamate release, we assessed eEPSCs recorded at +40 mV. We used two-way ANOVA with sex and treatment (saline or METH SA) as factors. Analyzing the amplitude of eEPSCs, we found a significant sex × treatment interaction (*F*_(1,38)_ = 6.5, *p* = 0.01; Fig. 3*A*), and *post hoc* Tukey’s analysis show that female METH rats have significantly larger eEPSC amplitude (*p* = 0.03) compared to female SAL. Figure 3*B* depicts representative traces of all groups. When analyzing the decay of eEPSCs, we found a significant sex by treatment interaction (*F*_(1,38)_ = 5.5, *p* = 0.02; Fig. 3*C*); however, *post hoc* examinations were not significant. We conducted planned contrasts (see Materials and Methods, Data analysis) and determined that male METH rats had significantly faster decay than saline counterparts (planned contrast, *t*_(20)_ = 2.3, *p* = 0.03). Similarly, we found a significant interaction when analyzing AUC (*F*_(1,38)_ = 5.5, *p* = 0.02; Fig. 3*D*) and planned contrast showed a significant increase in the AUC of METH females versus SAL females (*p* = 0.04). Analysis of the CV did not show significant differences (saline females: 0.16 ± 0.05; METH females: 0.20 ± 0.15; saline males: 0.18 ± 0.04; METH males: 0.26 ± 0.19; data not shown). In summary, METH SA significantly increased evoked glutamate in female rats and accelerated decay in males suggesting that METH SA increases NMDA currents in females without altering the kinetics of the NMDAR, whereas in males, METH SA affects NMDAR kinetics.

### GluN2B NMDAR blocker

**Figure 4.**
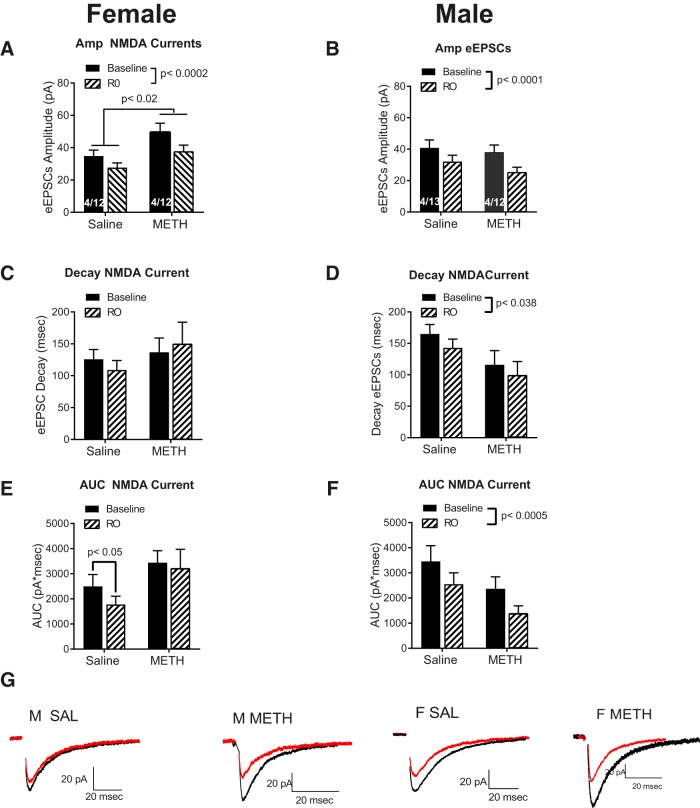
Effects of the GluN2B blocker. ***A***, Experiments performed in female rats show a main effect of treatment (SAL vs METH SA) and Ro256981 elicited a significant decrease in the NMDA current amplitude. ***B***, In male subjects, Ro256981 decreased the amplitude of NMDA currents, but there were no effects of treatment. ***C***, The GluN2B blocker did not affected the decay of the NMDA currents in females ***D***, In male rats, Ro256981significantly decreased the decay of NMDA currents. ***E***, The GluN2B blocker showed a trend for decreasing the AUC in saline female rats. ***F***, In male rats, Ro256981 significantly decreased the AUC. ***G***, Representative traces of the effects of Ro256981. Traces in black = baseline, traces in red = Ro256981. Each trace is the average of 10 events. Note the differences in scale.

In an effort to investigate the mechanisms mediating the effects of METH on eEPSCs, we assessed the role of the NMDA GluN2B subunit using Ro256981, a subunit-specific antagonist. We used repeated measures ANOVA with treatment (saline or METH SA) and test drug (baseline and Ro256981) as factors. We analyzed male and female data as separate ANOVAs due to the notable sex differences in sEPSCs and eEPSCs described before. In female rats (Fig. 4*A*), METH increased the amplitude of the NMDA currents relative to saline (main effect treatment; *F*_(1,16)_ = 6.27, *p* = 0.02) and Ro256981 reduced NMDA current amplitude relative to baseline (main effect test drug; *F*_(1,16)_ = 23.2, *p* = 0.0002). In male rats (Fig. 4*B*), Ro256981 decreased the amplitude of NMDA currents (main effect of test drug; *F*_(1,18)_ = 40.5, *p* = 0.0001), but there were no differences between METH and saline.

We also tested the effects of the GluN2B blocker on the kinetics of the NMDA currents. Ro256981 did not affect decay of NMDA currents in female rats (Fig. 4*C*), but interestingly, in male rats, Ro256981 significantly decreased NMDA current decay (main effect test drug, *F*_(1,18)_ = 5.0, *p* = 0.038; Fig. 4*D*).

Planned contrasts show that the GluN2B blocker decreased the AUC in female saline rats relative to baseline animals (planned contrast, *t*_(9)_ = 3.67, *p* = 0.05; Fig. 4*E*). Ro256981 was without an effect in METH females. In male rats, Ro256981 significantly decreased the AUC (main effect test drug; *F*_(1,18)_ = 17.16, *p* = 0.0005) regardless of METH or saline SA.

These results suggest that the METH SA-dependent female-specific increase in the amplitude of NMDA currents was not blocked by Ro256981. Furthermore, analysis of the kinetic data shows that only in female saline and males (SAL and METH) NMDA current decay was decreased by Ro256981. In summary, data suggest that the METH-mediated increase in the amplitude of eEPSCs in females was mediated by increases in GluN2B-lacking NMDA currents and confirm our previous report that METH SA elicits an increase in GluN2B-containing NMDAR in male rats.

## Discussion

To the best of our knowledge, this is the first report of sex differences in PFC synaptic activity in drug naïve males and females and those that have undergone METH SA. In this study, we found several sex differences: (1) female rats exhibit significantly lower basal amplitude of sEPSCs and faster kinetics than male rats, however, METH does not alter these measures in females or males; (2) METH SA increases the amplitude of eEPSCs in females; and (3) the GluN2B blocker Ro256981 significantly decreased the NMDA currents in males but did not block the METH-mediated increase in NMDA currents observed in female rats.

Females are reported to acquire METH SA faster than males (Roth and Carroll, 2004), take more drug (Roth and Carroll, 2004; Reichel et al., 2012; Bernheim et al., 2017), and have greater motivation to seek the drug (Roth and Carroll, 2004; Cox et al., 2013, 2017). Here, we used a METH SA protocol in which rats were given access to the drug for 6 h over 14 consecutive days. We do not report sex differences in drug intake, which is consistent with other studies using similar protocols (Johansen and McFadden, 2017; Venniro et al., 2017). However, female rats appear to take more drug on the first day of SA. Although this effect was not significant on a *post hoc* analysis, it is interesting to note that this day difference is consistent with reports that females acquire METH SA faster than males and take more drug than males (Roth and Carroll, 2004; Reichel et al., 2012; Bernheim et al., 2017). It is possible that enhanced METH intake during the 6-h session on day 1 rendered females more vulnerable to changes in PFC synaptic physiology discussed below. The overall lack of sex differences in drug intake render our sex differences in synaptic physiology particularly interesting because our behavioral output and drug exposure are equivalent yet the underlying neurophysiology are different. As such, we have identified a “convergent” sex difference in which behavioral endpoints are similar yet the underlying neurobiology is different (Becker and Koob, 2016).

### Baseline sex differences

to investigate sex differences in synaptic glutamatergic function, we explored the characteristic of sEPSCs and eEPSCs. Our results show that there are sex differences in baseline sEPSCs in the PFC, with female rats possibly exhibiting decreases in postsynaptic glutamate receptors and in excitatory strength. It is accepted that changes in amplitude of sEPSCs are associated with differences in the levels of postsynaptic receptors and/or changes in the dynamics of the receptors (Engelman and MacDermott, 2004; Costa et al., 2017) and changes in the kinetics of sEPSC events can be indicative of alterations in receptor subunit composition and their interactions with auxiliary subunits as well as alternative RNA splicing and post-translational modifications (Dingledine et al., 1999; Tomita, 2010; Granger et al., 2011; Stincic and Frerking, 2015). Our data show that female rats exhibit significant decreases in both parameters (i.e., amplitude and kinetics). Furthermore, since changes in sEPSC’s AUC are thought to represent the net charge transfer during an ionotropic glutamate receptor-mediated event, our results suggests that female animals have decreases in basal glutamatergic excitatory strength when compared to males (Keller et al.,1991).

To our knowledge, there have not been reports addressing basal sex differences in glutamate receptors or synaptic activity of the PFC, so the mechanisms underlying the differences in synaptic physiology are unknown.

### Sex differences and effects of METH treatment on synaptic glutamatergic activity

METH SA selectively increased the amplitude of eEPSCs in female rats. This result is supported by the significant increases found in the eEPSCs AUC in female rats. The AUC is thought to represent the net charge transfer, with increases indicating greater depolarization.

We have previously reported that METH SA increased GluN2B-containing NMDAR in males (Mishra et al., 2017). Here, we report again that METH SA elicits a significant reduction in the eEPSC decay in males compared to saline controls. METH-SA males show in average a decay of 120 ms (Fig. 3*C*) that it is closer to the typical decay reported for GluN1/2A NMDAR (∼40–50 ms; Monyer et al., 1992; Vicini et al., 1998; Wyllie et al., 1998; Yuan et al., 2009), rather than average decay reported for GluN1/2B NMDAR (300–400 ms); however: (1) since the kinetics of NMDAR have been characterized in non-neuronal expression systems, it is highly likely that the decay times recorded in pyramidal neurons will be very different, particularly given the potential for intracellular regulation of receptor kinetics; (2) since the NMDA currents vary widely across different types of neurons; and (3) since voltage clamp cannot clamp voltage along the neuron processes, and thus altered kinetics are expected, we propose that METH SA males exhibit a GluN1/2B NMDAR composition, replicating our previous results. Furthermore, we previously confirmed the subunit composition of NMDAR in METH SA males via tissue biotinylation and surface-protein expression.

In order to investigate whether METH SA increased GluN2B-containing NMDAR in females, we repeated those experiments. Interestingly, we found that the METH SA-dependent female-specific increase in amplitude of eEPSCs was not blocked by Ro256981, and analysis of the NMDA current kinetics shows that, in contrast to males, the GluN2B blocker did not affect the kinetics of NMDA currents in METH SA females.

While the mechanisms underlying METH-mediated increases in GluN2B-containing NMDARs are unknown, recently Li et al. (2017) published a report suggesting that in dopamine (DA) cells of the midbrain, amphetamine and METH either stimulate a population of extrasynaptic GluN2B-containing NMDARs or that it causes GluN2B-containing receptors to move laterally into the synapse. While further investigations will be needed to test this hypothesis, it is possible a similar mechanism regulates the increases in GluN2B-containing NMDARs in the PFC of males following METH SA. However, this hypothesis does not address the differences in METH-mediated effects between males and females. Further investigations examining NMDAR subunit composition, and their contributions to sex differences are needed. Interestingly, in male rats, we failed to detect a significant increase in sEPSC frequency following METH SA, in contrast to our previous findings (Mishra et al., 2017). We believe this difference is likely dependent on the amount of METH intake, as that was a critical difference between the two studies. It is possible that with longer METH SA experience (21 vs 14 d), this effect would be observed. It is of future interest to determine whether there is a threshold of METH quantity or a specific drug-experience pattern required to evoke this form of plasticity.

NMDARs are tetramers comprised of two obligatory GluN1 and two regulatory GluN2A or GluN2B subunits. Expression of the GluN2 subunit is developmentally regulated. GluN2B NMDARs are expressed in the developing brain and replaced by GluN2A NMDARs as the brain matures into adulthood (Sheng et al., 1994; Sans et al., 2000). GluN2A and GluN2B NMDARs differ with respect to biophysical properties (Vicini et al., 1998; Erreger et al., 2005; Hansen et al., 2014), synaptic localization (Townsend et al., 2003; Groc et al., 2006), activity-dependent trafficking (Barria and Malinow, 2002), and contributions to synaptic plasticity (Liu et al., 2004; Massey et al., 2004). Since the selective GluN2B blocker did not block the METH SA-mediated increases in female NMDA currents or affect their kinetics, we propose that, in contrast to males, in females, METH SA did not change the composition of the NMDAR. Thus, in male rats, METH SA seems to alter NMDAR subunit composition toward an immature state, but in female rats, it does not appear to alter adult NMDAR composition.

While our experiments did not investigate the subunit composition of NMDAR in male and female rats, it is tempting to speculate that since female METH SA rats do not show a strong presence of GluN2B NMDAR, the increase in NMDA current amplitude may be due to the phosphorylation of GluN2A, GluN2C, or GluN2D subunits in the female NMDAR. On the other hand, a study in alcohol-dependent rats has shown that GluN1 subunit protein levels in cerebral cortex increased 144 ± 12% compared to control levels in ethanol dependent female, but not male rats (Devaud et al., 1999), thus providing another possible target in the NMDAR that could be modified by METH SA.

The question remains, why does METH SA elicit a significant increase in NMDA currents in female but not male rats? In male rats, it has been shown that cortical activation of AMPAR increases extracellular levels of DA whereas activation of NMDAR decreases it (Jedema and Moghddam, 1996; Takahata and Moghaddam, 1998). These effects are mediated respectively by AMPARs preferentially activating VTA-projecting pyramidal neurons, resulting in an excitation of the VTA cells and increases in DA release, and via NMDAR activation of PFC interneurons resulting in inhibition of the cortico-VTA pathway, decreasing DA levels. A recent study has shown that, in contrast to males, activation of NMDAR increases DA levels in females (Locklear et al., 2016). Locklear and colleagues suggest that in females, NMDAR are activating mainly pyramidal neurons projecting to the VTA, thus eliciting an excitation instead of inhibition of VTA cells. The differences in NMDAR activation between male and females may be due to the effects that androgens exert, particularly in the PFC pyramidal cells projecting to the VTA (Aubele and Kritzer, 2012). Building on those findings, it is tempting to speculate that METH elicits an increase in DA release that in turns, in females, acts synergistically with the lack of androgen and results in an enhancement of NMDA currents.

## Conclusions

Our data show that female rats exhibit basic and evoked synaptic cortical differences when compared to males, and we propose that the mechanisms underlying the higher sensitivity of females to METH, as well as the faster acquisition of the drug by females, are mediated by these synaptic differences. We base this suggestion on our results showing that drug-naïve female rats exhibit significant decreases in amplitude and kinetics of sEPSCs compared to male rats but female rats with a history of METH SA exhibit higher amplitude of eEPSCs. Additionally, the GluN2B blocker Ro256981, which decreased NMDA currents in METH SA males, did not affect the NMDA currents in METH SA females. We speculate that the selective effects of METH in female rats may be due to the basal sex differences in the composition and sensitivity of NMDAR located in cortico-VTA projecting neurons.

Our experiments show that there are basal synaptic excitatory differences in PFC of males and female rats and these differences may explain some of the fundamental sex differences in addiction reported in the literature.

## Synthesis

Reviewing Editor: Karen Szumlinski, University of California at Santa Barbara Decisions are customarily a result of the Reviewing Editor and the peer reviewers coming together and discussing their recommendations until a consensus is reached. When revisions are invited, a fact-based synthesis statement explaining their decision and outlining what is needed to prepare a revision will be listed below. The following reviewer(s) agreed to reveal their identity: Matthew Hearing.

In consultation with the two original reviewers of your submission to eNeuro, we all agree that this revised manuscript is much improved. Reviewer 1 still expressed some reservations regarding the variability in the behavioral data for females but recognizes the importance of the physiological findings and deems the paper acceptable. Reviewer 2 still expresses concern regarding the claim that the ~120 ms decay kinetics align better with the receptor being GluN1/2B, rather than GluN1/2A. According to the citations that were included in the revision, I have to agree with Reviewer 2 that 120 ms is numerically closer to the typical decay kinetics of a GluN1/2A receptor (~40-50 ms), rather than the ~300-400 msec typically reported for GluN1/2B receptors. Given that the decay kinetics have been characterized in non-neuronal cells, it is highly likely that the decay times for any receptor expressed in non-neuronal cells is going to be different from what is observed in pyramidal neurons, particularly given the potential for intracellular regulation of receptor kinetics. We suggest that this fact be mentioned in the Discussion and I am happy to re-review for this revision. Also, Reviewer 2 noted that the traces in Figures 3 and 4 were missing segments so please ensure during the final publication stage that the traces are intact. To summarize, although both reviewers and I agree that there are some data in this report that should be followed up, the current report provides new information pertaining to sex differences in drug-induced neuroplasticity and warrants publication in eNeuro. Please find below the remaining concern of Reviewer 2 that I would like to see addressed in one more minor revision that will re-review prior to final acceptance.

Reviewer 2:

The authors state: “With the present data we cannot exclude the possibility that METH is increasing GluN2A containing receptors; however as can be observed in Fig 4, the decay for eEPSCs in saline control rates is in average 120 msec. The time constants describing the exponential deactivation time course (τdecay) are ∼40-50 ms for GluN1/2A, ∼300-400 ms for GluN1/2B and GluN1/2C, and ∼4 s for GluN1/2D (Monyer et al., 1992; Vicini et al., 1998; Wyllie et al., 1998; Yuan et al., 2009). Thus, our decay data are closer to the ones reported for GluN1/2B than to the ones reported for GluN1/2A. Furthermore, our previous findings reported in Mishra et al using tissue biotinylation and surface protein expression support our suggestion that METH SA increases GluN2B NMDAR.

The authors maintain that their decay kinetics align more with those expressing GluN1/2B however it's difficult to make that claim, as their mean decay is about 80 ms longer than GluN1/2A and ~180 ms shorter than GluN1/2B, which actually trends towards closer to GluN1/2A numerically speaking. Further, the references they provided were done in non-neuronal expression systems (HEK293, Oocytes), which makes it difficult to contend relevance to neurons, especially when the NMDA currents are so variable even across neuron types. Thus, while I personally remain unconvinced, given the little information on specific kinetics in L5/6 PYR in the PFC, as well as the arguments they provide and the current and past data, it seems perfectly fine to make their conclusions.

## Author Response

Rebuttal letter

Dear Dr Zsumlinsky:

We thank the editor and the reviewers by their careful revision and helpful comments. As a result, the actual manuscript has been improved.

We have made additions and changes to our manuscript (indicated by red font in one of the manuscript's versions) and below we address each one of the editor and reviewers comments in detail.

Editor and Reviewer 2:

1. In consultation with the two original reviewers of your submission to eNeuro, we all agree that this revised manuscript is much improved. Reviewer 1 still expressed some reservations regarding the variability in the behavioral data for females but recognizes the importance of the physiological findings and deems the paper acceptable. Reviewer 2 still expresses concern regarding the claim that the ~120 ms decay kinetics align better with the receptor being GluN1/2B, rather than GluN1/2A. According to the citations that were included in the revision, I have to agree with Reviewer 2 that 120 ms is numerically closer to the typical decay kinetics of a GluN1/2A receptor (~40-50 ms), rather than the ~300-400 msec typically reported for GluN1/2B receptors. Given that the decay kinetics have been characterized in non-neuronal cells, it is highly likely that the decay times for any receptor expressed in non-neuronal cells is going to be different from what is observed in pyramidal neurons, particularly given the potential for intracellular regulation of receptor kinetics. We suggest that this fact be mentioned in the Discussion and I am happy to re-review for this revision. Also, Reviewer 2 noted that the traces in Figures 3 and 4 were missing segments so please ensure during the final publication stage that the traces are intact. To summarize, although both reviewers and I agree that there are some data in this report that should be followed up, the current report provides new information pertaining to sex differences in drug-induced neuroplasticity and warrants publication in eNeuro. Please find below the remaining concern of Reviewer 2 that I would like to see addressed in one more minor revision that will re-review prior to final acceptance.

A1: we have added the following paragraph to the Discussion section: In here, we report again that METH SA elicits a significant reduction in the eEPSC decay in males compared to saline controls. METH-SA males show in average a decay of 120 msec (Fig 3C) that it is closer to the typical decay reported for GluN1/2A NMDAR (~40-50 ms, Monyer et al., 1992; Vicini et al., 1998; Wyllie et al., 1998; Yuan et al., 2009), rather than average decay reported for GluN1/2B NMDAR (300-400 msec), however: 1) since the kinetics of NMDAR have been characterized in non-neuronal expression systems, it is highly likely that the decay times recorded in pyramidal neurons will be very different, particularly given the potential for intracellular regulation of receptor kinetics, 2) since the NMDA currents vary widely across different types of neurons, and 3) since voltage clamp can't clamp voltage along the neuron processes, and thus altered kinetics are expected, we propose that METH SA males exhibit a GluN1/2B NMDAR composition, replicating our previous results. Furthermore, we previously confirmed the subunit composition of NMDAR in METH SA males via tissue biotinylation and surface-protein expression.

2. Reviewer 2 noted that the traces in Figures 3 and 4 were missing segments so please ensure during the final publication stage that the traces are intact.

A2: The traces are intact, the missing sections represent the stimulation artefact, that for clarity has been substituted by the black dot at the beginning of the traces.
